# Clinical and Biological Significance of *ESR1* Gene Alteration and Estrogen Receptors Isoforms Expression in Breast Cancer Patients

**DOI:** 10.3390/ijms20081881

**Published:** 2019-04-16

**Authors:** Anna Nagel, Jolanta Szade, Mariola Iliszko, Julia Elzanowska, Marzena Welnicka-Jaskiewicz, Jaroslaw Skokowski, Grzegorz Stasilojc, Jacek Bigda, Rafal Sadej, Anna Zaczek, Aleksandra Markiewicz

**Affiliations:** 1 Department of Medical Biotechnology, Intercollegiate Faculty of Biotechnology, University of Gdansk and Medical University of Gdansk, 80-211 Gdansk, Poland; anna.nagel@biotech.ug.edu.pl (A.N.); julia.elzanowska@gmail.com (J.E.); gstasilojc@gumed.edu.pl (G.S.); jjbigd@gumed.edu.pl (J.B.); rsadej@gumed.edu.pl (R.S.); azaczek@gumed.edu.pl (A.Z.); 2 Department of Pathology, Medical University of Gdansk, 80-210 Gdansk, Poland; jszade@gumed.edu.pl; 3 Department of Biology and Genetics, Medical University of Gdansk, 80-211 Gdansk, Poland; milisz@gumed.edu.pl; 4 Department of Surgical Oncology, Medical University of Gdansk, 80-211 Gdansk, Poland; mwelj@gumed.edu.pl; 5 Department of Oncology and Radiotherapy, Medical University of Gdansk, 80-210 Gdansk, Poland; jskokowski@gumed.edu.pl; 6 Department of Medical Laboratory Diagnostics -Biobank, Medical University of Gdansk, Gdansk, 80-210 Gdansk, Poland; 7 Biobanking and Biomolecular Resources Research Infrastructure (BBMRI.PL), 80-210 Gdansk, Poland

**Keywords:** breast cancer, estrogen receptor, ERα36, ERα66, gene amplification, prognostic factor

## Abstract

The amplification of estrogen receptor alpha (ERα) encoded by the *ESR1* gene has been described as having a prognostic role in breast cancer patients. However, increased dosage of the *ESR1* gene (tested by real-time PCR) is also observed in ER-negative breast cancers, which might suggest the expression of alternative isoforms of ERα (other than classical ERα of 66 kDa). In the current work, we have investigated the *ESR1* gene dosage in 402 primary breast cancer patients as well as the expression of ERα isoforms—ERα66 and ERα36—on mRNA and protein levels. The obtained results were correlated with clinicopathological data of the patients. Results showed that increased *ESR1* gene dosage is not related to *ESR1* gene amplification measured by fluorescent in situ hybridization (FISH), but it correlates with the decreased expression of *ERα66* isoform (*p* = 0.01). Interestingly, the short ER isoform *ERα36* was expressed in samples with increased *ESR1* gene dosage, suggesting that genomic aberration might influence the expression of that particular isoform. Similarly to *ESR1* increased gene dosage, high *ERα36* expression was linked with the decreased disease-free survival of the patients (*p* = 0.05), which was independent of the status of the classical *ERα66* level in breast tumors.

## 1. Introduction

Estrogen receptor alpha (ERα) is an important prognostic and predictive factor in breast cancer. It is a ligand-activated transcription factor and its signaling governs the growth, proliferation, and survival of cancer cells. This makes it a crucial target for endocrine therapies [1]. In breast cancer, nuclear ERα levels are routinely assessed by immunohistochemical methods particularly to determine the dependency of a tumour on estrogen-driven growth [2]; together with human epidermal growth factor receptor 2 (HER2), these are the basis of the molecular subtyping of breast cancers [3].

ERα is encoded by the *ESR1* gene located on chromosome 6. Due to its prognostic and predictive potential, *ESR1* gene alterations have been studied intensively. Gene amplification, as a mechanism of gene overexpression, may influence levels of the ERα protein, resulting in altered growth-stimulating signaling. Thus, changes in *ESR1* copy number are a subject of ever-present debate. Reported *ESR1* amplification rates in breast cancer range from 0% to 75% of patients [4,5,6,7,8,9]. Observed discrepancies have been related to different analytical techniques used for *ESR1* copy number analysis. We have previously developed a qPCR-based protocol measuring gene copy number alterations in topoisomerase IIα (*TOP2A*), which we showed was able to detect smaller structural changes in the *TOP2A* gene than large fluorescent in situ hybridization (FISH) probes [10]. We have applied a similar technique for the analysis of *ESR1* genomic sequence alteration [6]. With this method, we have shown that the *ESR1* copy number changes (gene dosage changes) occur also in ER-negative patients and have prognostic significance. In the current study, we aimed at exploring this seemingly paradoxical observation of absent (or low) ER protein level in the presence of increased *ESR1* gene dosage. Since the routine evaluation of ER in breast cancer focuses on the classical ER isoform of the molecular mass of 66 kDa (hence its name ERα66), aberrations in the *ESR1* gene were thus far correlated with levels of classical ER isoforms but omitted other ER isoforms. In 2005, Wang et al. described a 36-kDa splicing variant of the *ESR1* gene, which was called ERα36 [11,12]. It differs from the ERα66 isoform by lacking both transcriptional activation domains, but retains the DNA-binding domains [13,14], although new results indicate that it might act as a transcription factor [15]. Dissimilar to ERα66, which is usually detected in the cell nucleus, ERα36 localizes mainly to the cytosol and cell membrane [11,16], although nuclear localization is also observed [15,17,18]. ERα36 expression was described in ER-positive as well as in ER-negative breast cancer cell lines [17] and breast cancers [15,19,20]. The study performed by Lee et al. on 31 breast cancer patients describes that ERα36 assessed by immunohistochemical staining (IHC) is commonly expressed in breast cancers with different ER status [21]. A retrospective study of 896 breast cancer patients reveals that ERα66-positive patients with a high expression of ERα36 are less likely to benefit from hormonal therapy [19]. In vitro studies performed using various breast cancer cells indicate that ERα36 rapidly activates the MAPK signalling pathway, leading to uncontrolled proliferation and anti-apoptotic events [22].

Thus, we decided to explore whether the *ESR1* gene dosage measured by qPCR is related to the amplification of the whole *ESR1* gene and if it correlates with the expression of classical ERα66 as well as the short ER isoform ERα36. Additionally, the clinical significance of the measured parameters was assessed in the group of operable breast cancer patients.

## 2. Results

### 2.1. ESR1 Gene Dosage and Copy Number in Breast Cancers

**Figure 1:**
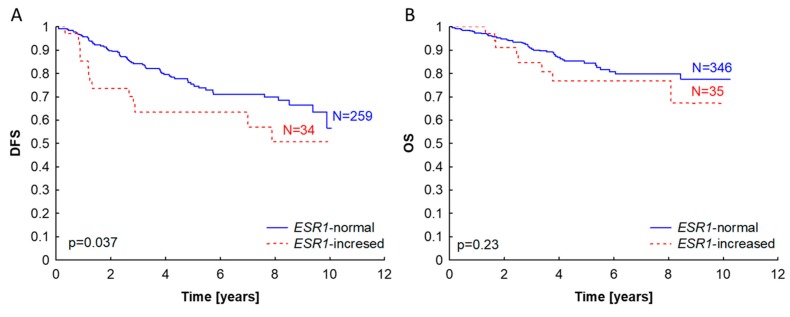
Kaplan–Meier survival curves according to *ESR1* gene dosage status (measured by qPCR) in primary breast tumors. *ESR1*-normal status was described as *ESR1/APP* ratio <2, *ESR1*-increased as *ESR1/APP* ratio ≥2. The probability of disease-free survival (**A**) and overall survival (**B**) are shown.

**Figure 2:**
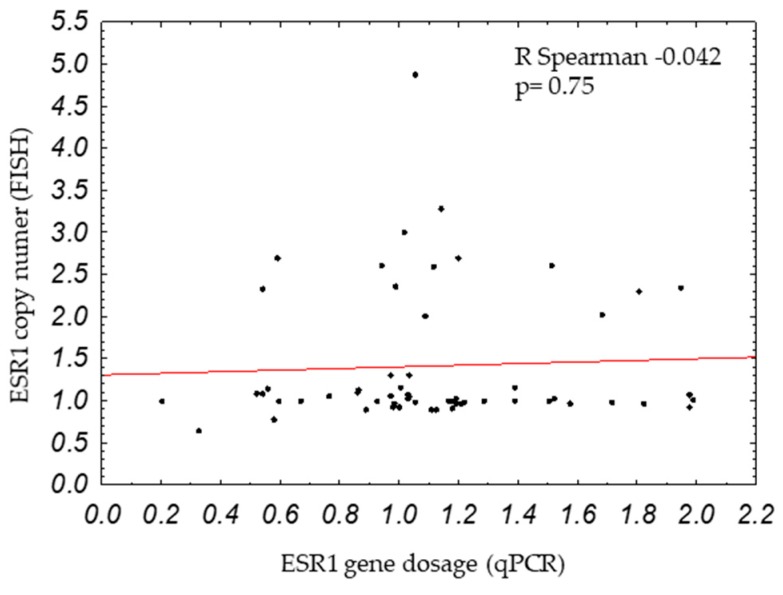
Correlation between *ESR1* copy number (measured by fluorescent in situ hybridization, or FISH) and *ESR1* gene dosage (measured by qPCR).

*ESR1* gene dosage was examined by qPCR in 402 samples of primary breast cancers. Increased *ESR1* gene dosage (*ESR1*/*APP* ratio ≥2) was observed in 9.2% (37/402) of the patients. As previously reported on a smaller group of patients [4], increased *ESR1* gene dosage was linked with the poor survival of the patients (Figure 1). As the literature describes the opposite findings, when *ESR1* gene status was tested by FISH, it made us wonder if increased *ESR1* gene dosage measured by qPCR would also correspond to *ESR1* amplification analyzed by the golden standard method for gene amplification testing: FISH. For this analysis, we selected 80 breast cancer samples that were additionally tested by FISH (successful results with both methods, qPCR and FISH, were available for 58 samples). In one case, polyploidy occurred with the average number of 3.5 CEN6 and 3.7 *ESR1* copies per cells; the sample was removed from the analysis. *ESR1* gene status measured by qPCR and FISH did not correlate (r = −0.042, *p* = 0.75, Figure 2), which suggests that both methods detect different types of alterations. FISH and qPCR assays detect different fragments in the *ESR1* gene: the FISH probe binds a region of 395 kbp, whereas with our qPCR assay, we detected an amplicon of 60 bp. Thus, FISH might detect larger, whole gene amplification, whereas qPCR might detect small changes in the *ESR1* gene that go undetected by the long FISH probe. Therefore, if *ESR1* gene dosage is not related to the amplification of the whole *ESR1* gene, we asked if *ESR1* gene dosage might be related to the altered expression of ER, including other than the classical ERα66 isoform, which could be the underlying factor conferring poor prognosis to breast cancer patients.

### 2.2. Genomic ESR1 Level versus mRNA and Protein Isoforms Expression

**Figure 3:**
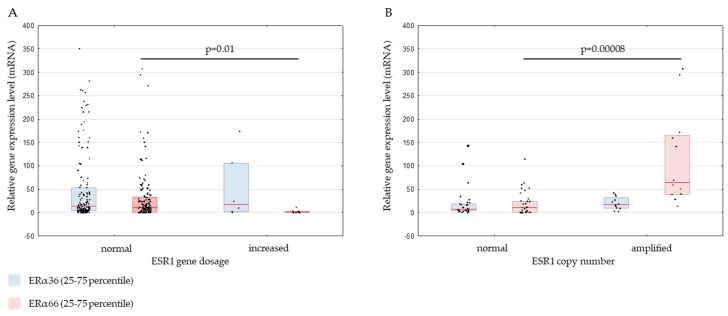
*ERα36* and *ERα66* relative gene expression levels in primary breast cancers classified according to *ESR1* gene dosage status measured by qPCR (**A**) or *ESR1* gene copy number measured by FISH (**B**). The vertical red line represents the median gene expression level. * *p* < 0.05, ** *p* < 0.0001. ERα: estrogen receptor alpha.

We have analyzed the expression of ERα36 and ERα66 on mRNA level by qPCR in frozen breast cancer samples and on protein level by IHC in formalin-fixed, paraffin-embedded (FFPE) samples. The results were correlated with *ESR1* gene tested by qPCR and FISH. On the mRNA level, reduced expression of the full-length ERα66 isoform was observed in samples with increased *ESR1* gene dosage (median relative expression 10.63 in *ESR1*-normal gene dosage and 0 in *ESR1*-increased gene dosage, *p* = 0.01; Figure 3A). On the contrary, increased ERα66 expression was found in samples with amplified *ESR1* measured by FISH (median gene expression level in *ESR1*-amplified samples [*ESR1*/*CEN*-6 ratio <2] −64.14 and in *ESR1*-normal samples [*ESR1*/*CEN*-6 ratio ≥2] −10.28; *p* = 0.00008, Figure 3B). However, there was no correlation between ERα36 expression and *ESR1* gene dosage (measured by qPCR; Figure 3A) or *ESR1* copy number (measured by FISH; Figure 3B). The expression of ERα36 was observed in samples with normal and increased *ESR1* gene dosage (Figure 3A), which also included ERα66-negative samples. However, depending on the *ESR1* gene dosage, the ratio of median ERα36 to ERα66 mRNA expression level (ERα36/ERα66) was markedly different: in *ESR1*-increased gene dosage samples, the ERα36/ERα66 ratio was 86, in comparison to *ESR1*-normal gene dosage samples, which had the ratio of 1.4. For *ESR1*-amplified and *ESR1*–normal samples by FISH, the ERα36/ERα66 ratio was almost unaffected (0.3 versus 0.7, respectively).

**Figure 4:**
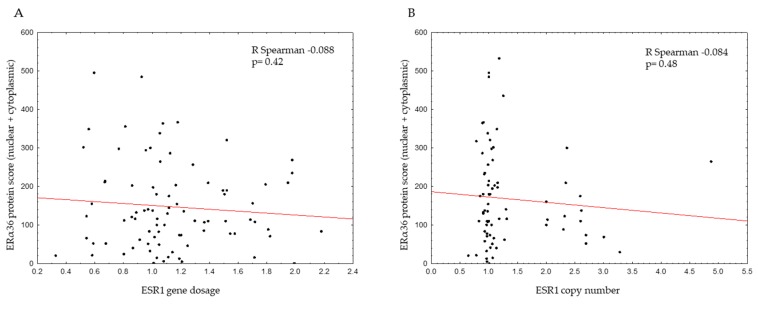
Correlation between ERα36 protein score (total nuclear and cytoplasmic score and *ESR1* gene dosage (measured by qPCR) (**A**) or *ESR1* copy number (measured by FISH) (**B**).

The *ESR1* gene dosage was also inversely correlated with ERα66 protein level (Table 1, *p* = 0.001) and mRNA expression (*p* = 0.0086; Table 1, Figure S3A). In samples with *ESR1*-increased gene dosage, 65% of samples were negative for ERα66 protein (according to the Allred score), whereas in samples with normal *ESR1* gene dosage, only 37% of the samples were ERα66-negative. Therefore, in ERα66-negative tumors, an *ESR1*-increased gene dosage was found in 15% (24/158) of the samples, in comparison to 5% (13/240) of the ER-positive samples. For *ESR1* gene status tested by FISH, there was a good positive correlation: all the *ESR1*-amplified tumors were ERα66-positive, and none of the ERα66-negative tumors carried *ESR1* amplification (*p* = 0.0038). *ESR1* gene copy number was also positively correlated with ERα66 gene expression (*p* = 0.00002; Table 1, Figure S3B). For ERα36 protein level (presented as the total IHC score: the sum of cytoplasmic and nuclear staining scores), no correlation was found between *ESR1* gene dosage or copy number (Figure 4A,B); similarly, there was no correlation between ERα36 mRNA expression and the *ESR1* gene by FISH and qPCR (Figure S3C,D). 

### 2.3. Correlation between ERα66 and ERα36 Isoforms on mRNA and Protein Level

**Figure 5:**
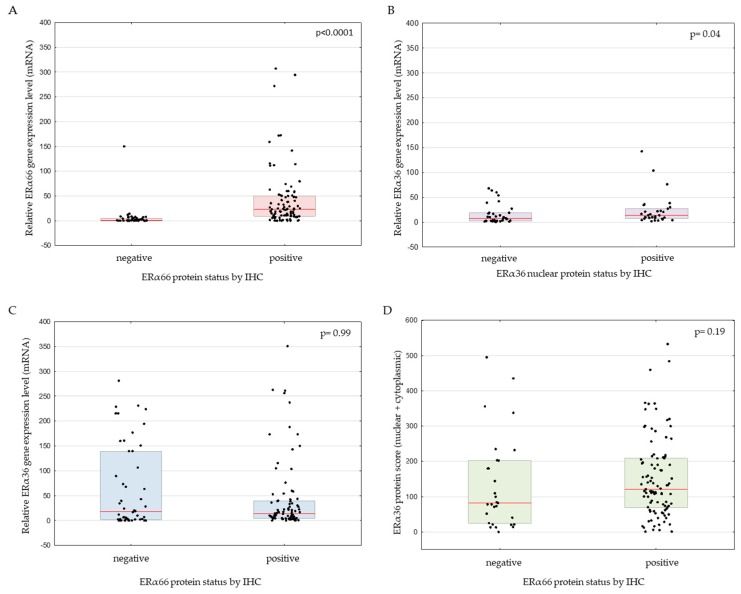
*ERα66* relative expression according to ERα66 immunohistochemical (IHC) protein status (**A**), *ERα3*6 relative expression according to ERα36 nuclear protein (**B**), *ERα3*6 expression according to ERα66 protein status (**C**), and ERα36 protein immunohistochemical score according to ERα66 protein status (**D**).

As expected, the level of ERα66 mRNA was increased in samples positive for ERα66 protein (*p* < 0.0001, Figure 5A); in the case of ERα36, correlation between mRNA and protein was observed, but only for the nuclear localization (*p* = 0.04, Figure 5B). Interestingly though, ERα36 mRNA was expressed on a similar level in IHC ERα66-negative and ERα66-positive samples (median relative gene expression levels of 17.8 and 13.7, respectively; *p* = 0.99, Figure 5C). Also on the protein level, ERα36 was not statistically different between ERα66-negative (median IHC score 82) and ERα66-positive samples (median IHC score 120; *p* = 0.19, Mann–Whitney test, *n* = 126, Figure 5D).

**Figure 6:**
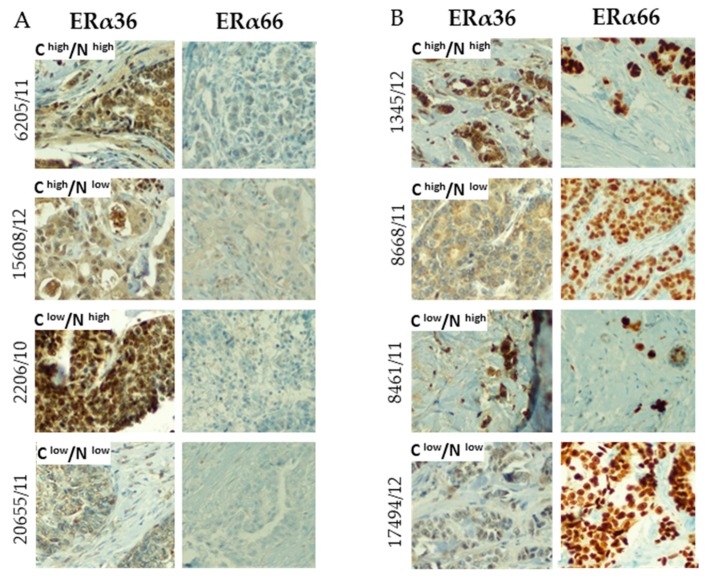
Exemplary photos of immunohistochemical staining of breast cancer samples with different ERα66 status (panel **A** – negative, panel **B** – positive according to Allred score) and ERα36 status. The ERa36 staining pattern has additionally been divided into positive and negative in cytoplasmic (**C**) and nuclear (N) localization based on the immunohistochemical score of the samples. All photos were taken under 20× magnification.

The ERα36 protein showed a different staining pattern than ERα66: whereas ERα66 was mostly located in the nucleus, ERα36 was observed both in the nucleus and cytoplasm, and also with different immunohistochemical scores in nuclear and cytoplasmic localization (Figure 6). The presence of ERα66 was not associated with the localization of the ERα36 isoform; both nuclear and/or cytoplasmic ERα36 was observed in ERα66-negative (Figure 6A) and positive samples (Figure 6B).

### 2.4. Clinical Significance of ERα Isoforms Expression

**Figure 7:**
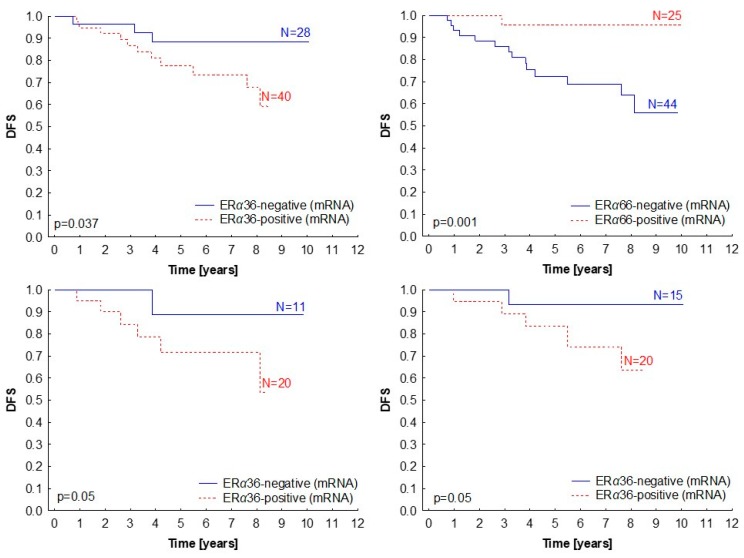
Kaplan–Meier survival curves presenting disease-free survival (DFS) according to *ERα36* (**A**) and *ERα66* (**B**) mRNA levels (measured by qPCR) in primary breast tumors. (**C**,**D**) Subgroup analysis for *ERα36* expression in ERα66 protein-negative (*p* = 0.05) (**C**) and positive (**D**) patients (*p* = 0.05). Median relative gene expression was a cut-off value for the classification of samples into negative and positive.

Since we have observed ERα36 isoform expression in patients with *ESR1* increased gene dosage and in ERα66-negative patients, we were interested to see how it influences the clinicopathological characteristics of the patients (Table 2). The ERα36 expression did not correlate with stage, grade, lymph node status, breast cancer molecular subtype, or histological tumor subtype, but its elevated expression (above median) was linked with shorter disease-free survival (*p* = 0.037; Figure 7A). Interestingly, ERα66 showed the exact opposite effect (*p* = 0.001; Figure 7B). Survival analysis in subgroups showed that the prognostic significance of the ERα36 isoform was sustained in both the ER-negative and ER-positive groups (Figure 7C,D), indicating that the ERα36 effect is independent of the presence of the full-length ERα66 isoform.

On the protein level, nuclear ERα36 was connected with decreased overall survival (*p* = 0.04), and cytoplasmic ERα36 showed a similar trend, but the result was not statistically significant (*p* = 0.12; Supplementary Figure S2). Data on disease-free survival (DFS) was not available for this set of patients.

### 2.5. The Role of ERα36 Isoform in Breast Cancer Cell Lines

**Figure 8:**
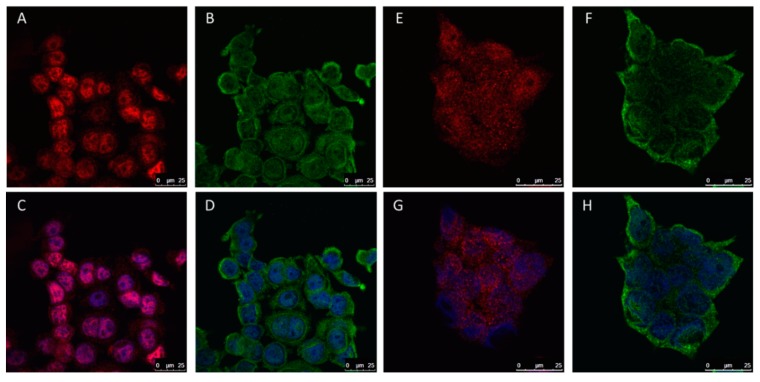
Immunofluorescent staining of two breast cancer cell lines: MCF7 (**A**–**D**) and BT474 (**E**–**H**). Staining for: ERα66 (red), ERα36 (green), and nucleus (blue).

Similarly to clinical samples, we observed that in ERα66-positive cell lines, the MCF7 and BT474, ERα36 isoform localized independently from ERα66. ERα36 was found in the cytosol and nucleus, whereas ERα66 was found predominantly in cell nucleus (Figure 8).

**Figure 9:**
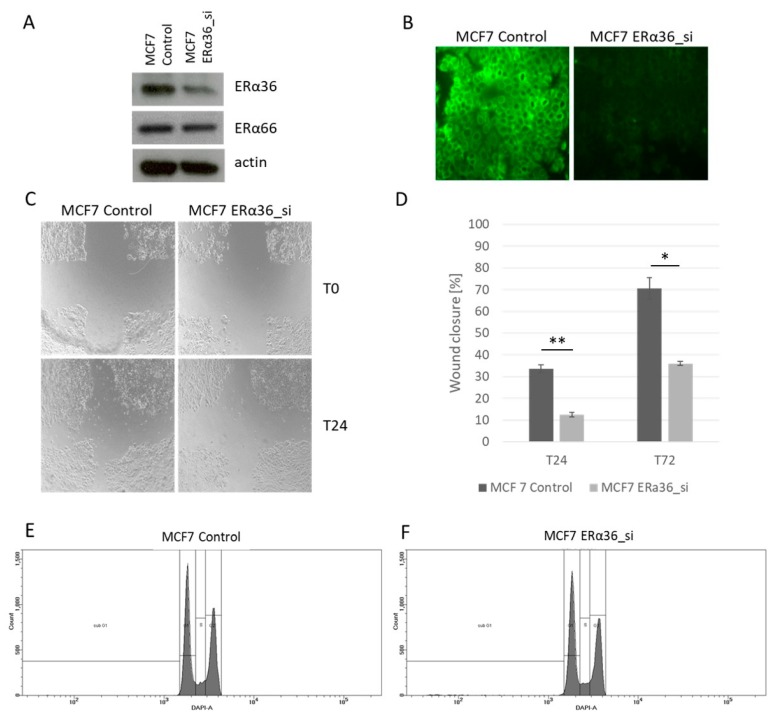
Silencing the ERα36 by siRNA confirmed by Western blotting (**A**) and immunofluorescence (**B**). Decrease in cell migration measured by wound healing assay in cells with silenced ERα36 (**C**,**D**, * *p* = 0.005, ** *p* = 0.002). Cell cycle analysis by flow cytometry in the MCF7 wild-type cell line (MCF7 Control) and with ERα36 silenced (MCF7 ERα36_si) showed no differences in the number of cells in a given cell cycle phase (**E**,**F**).

Basing on our observations from the clinical samples, where the prognostic significance of ERα36 was observed, we asked if silencing ERα36 will translate to decreased migration and changes in the cell cycle of breast cancer cell lines. Three siRNAs against ERα36 were tested, and siRNA#3 was chosen as it resulted in the strongest decrease in ERα36 protein level measured by Wester blot (Figure 9A,B). As a negative control, non-targeting siRNA was used in all the experiments. MCF7 cells with silenced ERα36 showed a significantly lower ability to migrate in wound-healing assay (Figure 9C,D). Silencing had no effect on cell cycle analyzed by flow cytometry (Figure 9E,F).

## 3. Discussion

As the estrogen receptor is one of the most important molecular targets in breast cancer, it is crucial to deeply understand every aspect of its functioning, as it might help to predict treatment response or disease course. The amplification of *ESR1* was described in mastopathic breast tissue, which progressed to invasive cancer [23] or pre-malignant endometrial cancers [24], implying early *ESR1* amplification and its role in carcinogenesis. Moreover, the amplification of *ESR1* was shown to be linked with good prognosis in tamoxifen-treated breast cancer patients [4,25]. Nevertheless, large variations are reported in the frequency of *ESR1* amplification in breast cancer (0–75%, reviewed in [9]), depending on the methodology and applied cut-off values. Regarding the correlation between *ESR1* amplification and the expression of ER, studies results are divided. As a general rule, in morphology-guided methods of *ESR1* amplification analysis, such as FISH, correlations between gene and protein levels were more frequently observed [4,5,23,26,27]. It is still being debated if the analysis of *ESR1* amplification assessed by FISH can be influenced by the FISH probe binding to the *ESR1* mRNA, generating false-positive signals [5,7,28]. In our analysis, we have used validated FISH probes, which give similar results (*ESR1* copy number) with and without RNase treatment [5], making RNase treatment a dispensable step.

We have previously reported that increased *ESR1* gene dosage measured by qPCR has prognostic significance in breast cancer patients [6]. Now, we have performed additional *ESR1* gene dosage analysis using the previously developed qPCR method. We observed increased *ESR1* gene dosage (*ESR1/APP* ratio ≥2) in 9.4% of all patients. Interestingly, *ESR1* gene dosage increase was observed more frequently in ER-negative compared to ER-positive patients (15% versus 5% of the patients). Since gene amplification is a known mechanism for protein overexpression, one would expect the biological effect to be observed only in patients with ER-positive tumors. Therefore, we have selected a subset of sample for which *ESR1* gene status was measured both with qPCR and a golden standard method: FISH. We have shown that there is no correlation between *ESR1* gene dosage (measured by qPCR) and *ESR1* copy number (measured by FISH), which underlines that qPCR detects different changes than FISH. We had a similar observation when analyzing changes in the topoisomerase II α gene (encoded by *TOP2A*) in breast cancers [29]. Possibly, much smaller changes are detected by qPCR than by large FISH probes spanning the whole gene sequence [10]. This would make the aberrations measured by qPCR go undetected by large FISH probes.

To our knowledge, this is the first study describing aberrations in the *ESR1* gene in the context of the expression of ER isoforms. In the current work, we have tested the expression of two ER isoforms on mRNA and protein levels: classical ERα66 and a short variant, ERα36, which was recently described as playing a role in breast cancer aggressiveness [11]. Regarding the expression levels of isoforms, *ESR1*-increased gene dosage measured by qPCR was linked with decreased ERα66 expression (on the mRNA and protein level), as opposed to *ESR1* amplification measured by FISH, which correlated with increased ERα66 protein and mRNA. In the case of the *ERα36* isoform, no correlation was observed between *ESR1* measured by qPCR or FISH. The qPCR amplicon, which is designed to detect changes in *ESR1* gene dosage, is located in exon 1 of the *ESR1* gene. Since small aberrations in regulatory regions might affect the expression level of a gene or its splice variants [30], it is possible that the *ESR1* gene dosage alterations that we have measured by qPCR decrease the ERα66 level, but do not change ERα36 expression. Indeed, in samples with *ESR1*-increased gene dosage, the ERα36/ERα66 ratio was over 60 times higher than that in the *ESR1*-normal samples. Therefore, we have investigated what is the prognostic role of ER isoforms, being especially interested in the novel ERα36, for which data is very limited. We observed that the two investigated isoforms have a dramatically different impact on survival. Whereas classical ERα66 is a marker of good prognosis, ERα36 was related to decreased disease-free survival, which is similar to the increased *ESR1* gene dosage measured by qPCR. The prognostic effect of ERα36 was visible in both ERα66-negative and ERα66-positive patients. This points to ERα66-independent mechanism of ERα36 action; even in samples with high ERα66 expression, ERα36 still conferred poor prognosis. This confirms the results by Wang et al., who also reported the negative influence of ERα36 expression on the survival of both ERα66-negative and ERα66-positive breast cancer patients, especially those treated with tamoxifen [15]. The study of Shi et al. also showed the prognostic role of ERα36, but only in ERα66-positive patients treated with tamoxifen (and not in ERα66-negative patients) [19]. Further studies are required to uncover the dependence between ERα isoforms and their impact on the survival of breast cancer patients during treatment.

Other groups have reported on the expression of the ERα36 isoform in breast cancer cell lines [11,17,20,31,32,33,34] and breast cancer patients [15,19,20,34,35,36]. Deng et al. [31,32] as well as Yin et al. [33] showed that increased ERα36 expression is important for the maintenance of the stem cell population in breast cancers, and Zhao indicated its role in tamoxifen resistance, which is possibly related to non-genomic signaling via the MAPK and Akt pathways [34]. On the other hand, Wang et al. proposed a mechanism in which after binding to estrogen or Tamoxifen, ERα36 translocates to the nucleus and acts as a transcription factor for stem cell marker *ALDH1A1* [15]. The tamoxifen resistance of the MCF7 cell line was also attributed to the role of ERα36 in the downregulation of ERα66 [37]. Also, Zhang et al. described an inversed association between the expression of ERα36 and ERα66 isoforms [17]. We have not observed any correlation between the expression of the two ER isoforms, but this effect might be specific to the tumor cell type and state (such as the condition of acquired tamoxifen resistance). In the in vitro functional tests, we showed that the silencing of ERα36 decreased the migration of the MCF7 breast cancer cell line, which was also described by Li et al. [37]. Thus, ERα36 might be important for the invasive potential of cancer cells, which translates to the poor outcome of the patients. 

## 4. Materials and Methods

Four hundred and eighteen primary tumours from breast cancer patients (stages I–IV) treated in the Medical University of Gdansk were investigated. Their detailed clinical characteristics are listed in Table 3. The study was granted permission from the Bioethical Committee of the Medical University of Gdansk. *ESR1* gene dosage was tested using qPCR on frozen primary tumours, the data was successfully obtained for 402 samples. *ESR1* gene amplification was measured using fluorescent in situ hybridization (FISH) on tissue microarrays containing 80 formalin-fixed, paraffin-embedded (FFPE) tissues samples. For *ERα36* and *ERα66* gene expression analysis, 149 frozen primary tumor samples were used. The median age of the patients was 58 years (27–61 years). Informed consent was collected from all the participants who were included in the study.

### 4.1. RNA and DNA Isolation, Reverse Transcription, and Gene Expression Analysis

Primary tumor samples were collected during surgery, snap frozen, and stored at –80 °C. DNA and RNA isolation was performed usingan AllPrep DNA/RNA Kit (Qiagen, Hilden, Germany) according to the manufacturer protocol. Briefly, 30 mg of frozen tissue was lysed in lysis buffer and ceramic beads using a MagNA Lyser (Roche, Basile, Switzerland), then added to the DNA-binding column and processed further for DNA isolation. Flow-through was used to isolate RNA with the DNase digestion step. Purified RNA (1000 ng) was reverse-transcribed with random hexamers to cDNA using a Transcriptor First Strand cDNA Synthesis Kit (Roche, Basil, Switzerland) and Mastercycler Gradient Thermal Cycler (Eppendorf, Hamburg, Germany) according to the manufacturer protocol. The expression of *ERα36* and *ERα66* was measured using real-time PCR with Universal PCR mastermix (Applied Biosystems, Foster City, CA, USA), giving 10 ng of cDNA per reaction. Two reference genes *GAPDH* and *YWHAZ* were chosen based on their expression stability in the tested samples. Sequences of the primers were as follows. For *ERa36:* forward, 5′-CCAAGAATGTTCAACCACAACCT-3′; reverse, 5′-GCACGGTTCATTAACATCTTTCTG-3′. For *ERα66*: forward, 5′-AAGAAAGAACAACATCAGCAGTAAAGTC-3′; reverse, 5′-GGGCTATGGCTTGGTTAAACAT-3′. For *GAPDH:* forward, 5′-ACAACTTTGGTATCGTGGAAGG-3′; reverse, 5′-GCCATCACGCCACAGTTTC-3′. For *YWHAZ,* forward, 5′-TGTAGGAGCCCGTAGGTCATC-3′; reverse, 5′-GTGAAGCATTGGGGATCAAGA-3′. The PCR programme used was: 95 °C for 10 min; then, 40 cycles of 95 °C for 15 s, 60 °C for 1 min, and 95 °C for 10 s. The melting curve was performed by monitoring fluorescence in the samples, which were heated up from 65 °C to 95 °C in 0.5 °C increments.

### 4.2. ESR1 Gene Dosage Analysis with qPCR

*ESR1* gene dosage in frozen primary tumors was tested according to our method described before [6]. Briefly, *ESR1* gene dosage was measured in a relative manner (to *APP* reference gene and full blood as a calibrator) in real-time PCR (CFX96 cycler, Bio-Rad, Hercules, CA, USA) with Locked Nucleic Acid hydrolysis probes. Gene dosage equal to or higher than two was defined as *ESR1* increased gene dosage.

### 4.3. ESR1 Copy Number Analysis with Fluorescent in situ Hybridization

FISH analysis was performed on tissue microarray (TMA) sections of FFPE samples from 80 breast cancer patients using a ZytoLight SPEC ESR1/CEN 6 Dual Color Probe (ZytoVision, Bremerhaven, Germany) and ZytoLight FISH-Tissue Implementation Kit (ZytoVision, Bremerhaven, Germany) according to the guidelines of the manufacturer. The evaluation of fluorescent signals was performed by the analysis of images under the fluorescent microscope. Twenty to thirty nuclei cells were evaluated per sample; the number of fluorescent signals from the *ESR1* probe and centromere 6 (CEN6) probe was counted, averaged, and converted into a ratio of *ESR1/CEN6* signal per cell. The *ESR1/CEN6* ratio of ≥2 was classified as *ESR1* amplification, while the *ESR1/CEN6* ratio between 1.3–2 was classified as gain (according to [5]). Exemplary photos of breast cancer samples analyzed for *ESR1* gene amplification with FISH are shown in Figure S1.

### 4.4. ERα36 Protein Level Analysis with Immunohistochemistry

Tissue microarrays (TMA) were prepared by sampling up to five non-adjacent tissue cores of 1-mm diameter from each FFPE primary tumor. Serial sections were analyzed by manual immunohistochemical staining with commercially available rabbit antibodies against ERα36 specific to unique C-terminal sequence (Cell Applications Inc. San Diego, CA, USA, Cat# CY-1109; dilution 1:800, incubation time 1 h). Secondary anti-rabbit antibodies conjugated with horseradish peroxidase (HRP) were used together with the Novolink Max-Polymer Detection System (Leica Novocastra, Wetzlar, Germany) for the detection of the ERα36 protein. Intensity (scale 0–3) and the percentage of positively stained cells were evaluated in the nucleus and cytoplasm/membrane, giving an immunohistochemical score between 0–300. The results of the immunochistochemical staining of FFPE breast cancer samples with low and high immunohistochemical scores and the Western blotting analysis of the corresponding frozen breast cancer samples are presented in the Supplementary Figure S5.

### 4.5. ERα66, PR, and HER2 Status Analysis in Breast Cancer Samples

Hormone receptor status in breast cancer samples was assessed by IHC with mouse monoclonal antibody against ERα66 (clone 1D5, Dako Agilent, Santa Clara, CA, USA) and PR (clone 636, Dako Agilent, Santa Clara, CA, USA) according to the manufacturer’s instructions. Antibodies were used at a dilution of 1:50; antigen retrieval was performed at neutral pH by water-bath heating at 90 °C for 30 min. Visualization was performed with the Envision Dako (Dako Agilent, Santa Clara, CA, USA) system. For ER and PR, evaluation of the immunohistochemical nuclear staining was performed based on an Allred score or the immunoreactivity of any intensity in at least 10% of the tumor cells (for older tumor samples). HER2 receptor status was analyzed by IHC: a 3+ score was considered positive, and 2+ cases were equivocal and were tested for HER2 gene amplification with FISH with the use of PathVision HER2 DNA Probe Kit (Abbott Molecular, Abbott Park, IL, USA), according to the manufacturer’s instructions. The mean numbers of HER2 and centromer 17 signals were estimated for each tumor sample. A ratio of HER2/CEP-17 ≥2 was considered as HER2 amplification and an HER2-positive result.

### 4.6. Cell Culture

MCF7 and BT474 cells were purchased from the American Tissue Culture Collection (ATCC, Manassas, VA, USA). Cells were passaged for a maximum of 3 to 4 months post resuscitation and routinely tested for mycoplasma contamination. MCF7 cells were cultured in DMEM supplemented with 10% fetal bovine serum (FBS). BT474 cells were maintained in RPMI-1640 with 10% FBS and 5 μg/mL insulin. All the media and their supplements were from Sigma-Aldrich (Saint Louis, MO, USA) or HyClone (GE Healthcare, Chicago, IL, USA).

### 4.7. Gene Knock-Down with siRNA and Western Blotting

Cells were seeded and transfected after 24 h using Lipofectamine 2000 according to the producers’ protocol. The siRNA used in this experiment was designed to interfere with ERα36 mRNA. The following sequences were chosen: sense 5′-AUGCCAAUAGGUACUGAA-3′ and antisense 5′-UUCAGTACCUAUUGGCAU-3′. We confirmed gene silencing in the MCF7 cell line by Western blotting. Cell lysate was prepared using RIPA buffer (Sigma Aldrich, Saint Louis, MO, USA), while protein concentration was measured by a BCA assay kit (Thermo Fisher Scientific, Waltham, MA, USA). Proteins were separated using 12% polyacrylamide TGX gels (Bio-Rad, Hercules, CA, USA) and transferred onto the PVDF membrane by semi-dry transfer (Bio-Rad, Hercules, CA, USA). For detection, we used primary rabbit anti-ERα36 antibody (Cell Applications Inc. San Diego, CA, USA; dilution 1:500), mouse anti-ERα66 antibody (SantaCruz, Dallas, TA, USA; dilution 1:1000), and anti-β-actin antibody (Sigma Aldrich, Saint Louis, MO, USA; dilution 1:10,000). Appropriate, secondary anti-rabbit and anti-mouse HRP-conjugated antibodies were used (Sigma Aldrich, Saint Louis, MO, USA; dilution 1:100,000).

Analysis of ERα36 levels in frozen breast cancer samples with Western blotting was performed according to the protocol described above, apart from the homogenization step, where frozen tumor samples were cut and suspended in RIPA buffer (Sigma Aldrich, Saint Louis, MO, USA); then, tissue was minced using a sterile scalpel and centrifuged at 10,000× *g* for 10 min in 4 °C. Homogenization was followed by the measurement of protein concentration by a BCA assay kit (Thermo Fisher Scientific, Waltham, MA, USA).

### 4.8. Immunofluorescent Staining

Cells were seeded on the sterilized cover glass and after 24 h were fixed and permeabilized using a methanol/acetone mix for 15 min. For blocking, 5% BSA in PBS was used. Primary antibodies were diluted in Antibody Diluent (Dako Agilent, Santa Clara, CA, USA) and incubated with cells for 30 min. The following antibodies were used: rabbit anti-ERα36 (Cell Applications Inc. San Diego, CA, US, dilution 1:1000) and mouse anti-ERα clone 1D5 (Dako Agilent, Santa Clara, CA, USA; dilution 1:400). As secondary antibodies, appropriately anti-rabbit IgG DyLight 488 and anti-mouse IgG DyLight 594 were used (Thermo Fisher Scientific, Waltham, MA, USA; dilution 1:2000).

### 4.9. Wound-Healing Assay

Cells were seeded into non-coated 24-well plates in full medium (DMEM with 10% serum). Wounds were made with a sterile pipette tip after cells reached confluence, and cell debris was washed; then, incubation was performed in DMEM medium with 2% serum in order to inhibit cell proliferation. Wound closure was measured after 24 h using a phase-contrast microscope. Data was analyzed using the “MRI Wound Healing” ImageJ plugin, and statistical analyses (*t*-test) were performed using MS Office Excel software.

### 4.10. Cell Cycle Analysis

Cells were seeded on the six-well plates, and after 24 h, siRNA was added to appropriate samples. After 48 h, cells were harvested washed with PBS and fixed with 70% ice-cold ethanol for 30 min. After the depletion of ethanol, the mixture of 10 µg/mL DAPI and 2 mg/mL RNase free from DNases in PBS was added for 15 min. Then, cells were analyzed using a LSR II flow cytometer (BD Biosciences, Franklin Lakes, NJ, USA).

### 4.11. Statistical Analysis

All the analyses were performed using Statistica version 12 (StatSoft Dell, Round Rock, TA, USA) software. Categorical variables were compared by χ2 test. Continuous variables were compared by Spearman’s rank order test. The Mann–Whitney test was used to examine the differences between continuous values in two groups. Kaplan–Meier curves for disease-free survival were compared using an F-Cox test.

## 5. Conclusions

We have shown that increased *ESR1* gene dosage is found more frequently in ERα66-negative patients. The lack of a full-length ER isoform, ERα66, did not preclude the expression of a short ERα36 variant. The expression of both isoforms was related to patients’ survival, but their effect was opposite—high ERα66 levels conferred good prognosis, whereas high ERα36 conferred poor prognosis. The obtained results underline the complexity of molecular networks in which estrogen receptor is involved. Together with other findings linking the expression of ERα36 to resistance to endocrine therapies, the role of alternative ER isoforms should be further investigated in order to uncover their biological function and clinical utility.

## Figures and Tables

**Table 1:** Correlation between ERα66 protein status, ERα66 gene expression, and *ESR1* gene status measured by qPCR or FISH.

**Header**	***ESR1*** **Gene Dosage Status (qPCR)** **[Number of Cases (%)]**		**Total**	***p***	***ESR1*** **Gene Copy Number (FISH)** **[Number of Cases (%)]**		**Total**	***p***
	**Normal**	**Increased**			**Normal**	**Amplified**		
**ERα66 Protein Status**								
negative	134 (37%)	24 (65%)	158	0.001	21 (36%)	0	21	0.0038
positive	227 (63%)	13 (25%)	240		37 (64%)	15 (100%)	52	
Total	361 (100%)	37 (100%)			58 (100%)	15 (100%)	73	
	***ESR1*** **Gene Dosage Status (qPCR)**		**Total**	***p***	***ESR1*** **Gene Copy Number (FISH)**		**Total**	***p***
	**Normal**	**Increased**			**Normal**	**Amplified**		
ERa66 mRNA Expression, Median (25–75 Percentile)	10.63 (0.84–33.1)	0.21 (0–3.22)	145	0.0086	10.28 (0.2–24.5)	64.14 (39–165.3)	45	0.00002

**Table 2:** Correlation between ERα36 relative expression level (measured by qPCR) and the clinicopathological characteristics of the patients. HER2: human epidermal growth factor receptor 2.

Variable	N	Median ERα36 Expression (25–75th Percentile)	*p*
T stage			*p* = 0.41
T1–2	127	13.17 (3.75–42.90)	
T3–4	10	58.02 (7.37–160.88)	
N stage			*p* = 0.69
N0	78	17.20 (3.52–63.44)	
N1	58	9.95 (4.23–40.05)	
Grading			*p* = 0.92
1	13	18.84 (5.34–27.19)	
2	63	9.89 (3.75–37.41)	
3	42	9.97 (2.89–39.08)	
Histological subtype			*p* = 0.21
Ductal	54	31.28 (4.1–144.7)	
Lobular	7	74.39 (1.1–180.7)	
Other	4	162.13 (78.1–205.5)	
Molecular type			*p* = 0.84
Luminal A	17	16.70 (4.68–150.35)	
Luminal B HER2–	13	11.18 (3.98–24.24)	
Luminal B HER2+	2	97.21 (0–194.42)	
Non luminal HER2+	6	83.57 (0–160.88)	
Triple negative	13	39.56 (2.88–160.1)	
ER status			*p* = 0.99
0	50	17.84 (1.91–139.11)	
1	87	13.71 (4.41–39.08)	
PR status			*p* = 0.08
0	52	10.46 (1.52–53.17)	
1	85	15.77 (5.34–54.09)	
HER2 status			*p* = 0.35
0	104	13.82 (3.94–59.45)	
1	24	10.41 (2.56–31.21)	

**Table 3:** Patient characteristics.

Variable	Number of Cases (%)	
**Age**		
<50	120	(29)
>50	298	(71)
**T Stage**		
1	140	(33)
2	194	(46)
3	40	(10)
4	39	(9)
Missing data	5	(2)
**N Stage**		
negative	207	(49)
positive	206	(49)
Missing data	5	(2)
**Grade**		
1	30	(7)
2	171	(41)
3	135	(32)
Missing data	82	(20)
**Histologic Type**		
Ductal	218	(52)
Lobular	54	(13)
Other	26	(6)
Missing data	120	(29)
**ER Status**		
negative	164	(39)
positive	250	(59)
Missing data	4	(2)
**PR Status**		
negative	175	(42)
positive	239	(57)
Missing data	4	(2)
**HER2 Status**		
negative	274	(66)
positive	59	(14)
Missing data	85	(20)

## Supplementary Materials

The following are available online at https://www.mdpi.com/1422-0067/20/8/1881/s1. Figure S1: *ESR1* gene copy number analysis with by fluorescent in situ hybridization (FISH). Figure S2. Prognostic significance of ERa36 on protein level. Kaplan–Meier survival curves presenting overall survival (OS) according to ERα36 protein level in the nucleus (A) and cytoplasm (B). Division into positive/negative samples was made based on median immunohistochemical score (percentage of positively stained cells x intensity of the staining). Figure S3. Correlation between *ERα66* (A,B) and *ERα36* (C,D) mRNA expression and *ESR1* gene status. *ESR1* gene dosage assessed by qPCR (A, C) and ESR1 copy number assessed by FISH (B, D). Figure S4: Correlation between nuclear (A, B) as well as cytoplasmic (C, D) ERα36 levels and ERα66 assessed by immunohistochemistry. ERα36 levels are presented as either a percentage of positive cells (A, C) or immunohistochemical score (percentage of positive cells (0–100%) x staining intensity (0–3); B, D). Figure S5: ERα36 protein levels assessed by Western blot in frozen breast cancer samples and matched FFPE samples using immunohistochemistry. ERα36 low – samples with low total ERα36 immunohistochemical score; ERα36 high – samples with high total ERα36 immunohistochemical score.

## Abbreviations

ESR1	estrogen receptor gene
ER	estrogen receptor alpha
ERα36	estrogen receptor alpha, 36kDa isoform
ERα66	estrogen receptor alpha, 66kDa isoform
FISH	fluorescent in situ hybridization
HER2	human epidermal growth factor receptor
IHC	immunohistochemistry
PR	progesterone receptor alpha
OS	overall survial
DFS	disease free survival
