# Supplementary figures and images for: Clinical and Biological Significance of ESR1 Gene Alteration and Estrogen Receptors Isoforms Expression in Breast Cancer Patients
Source: Int J Mol Sci. 2019 Apr 16;20(8):1881. doi: 10.3390/ijms20081881 (PMC6514554; doi:10.3390/ijms20081881)

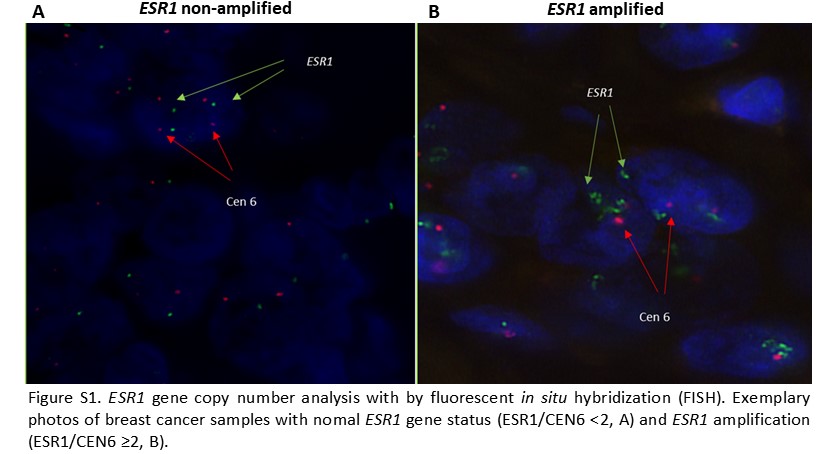

Supplement: Supplementary file 1 [file ijms-20-01881-s001.zip › Supplemantary data/Figure S1.jpg]

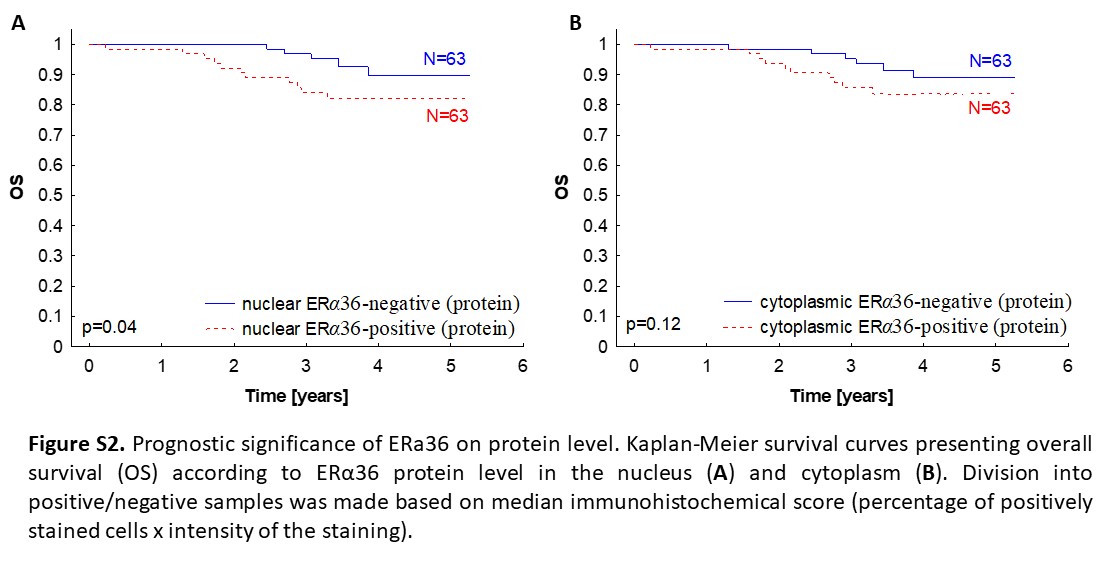

Supplement: Supplementary file 1 [file ijms-20-01881-s001.zip › Supplemantary data/Figure S2.jpg]

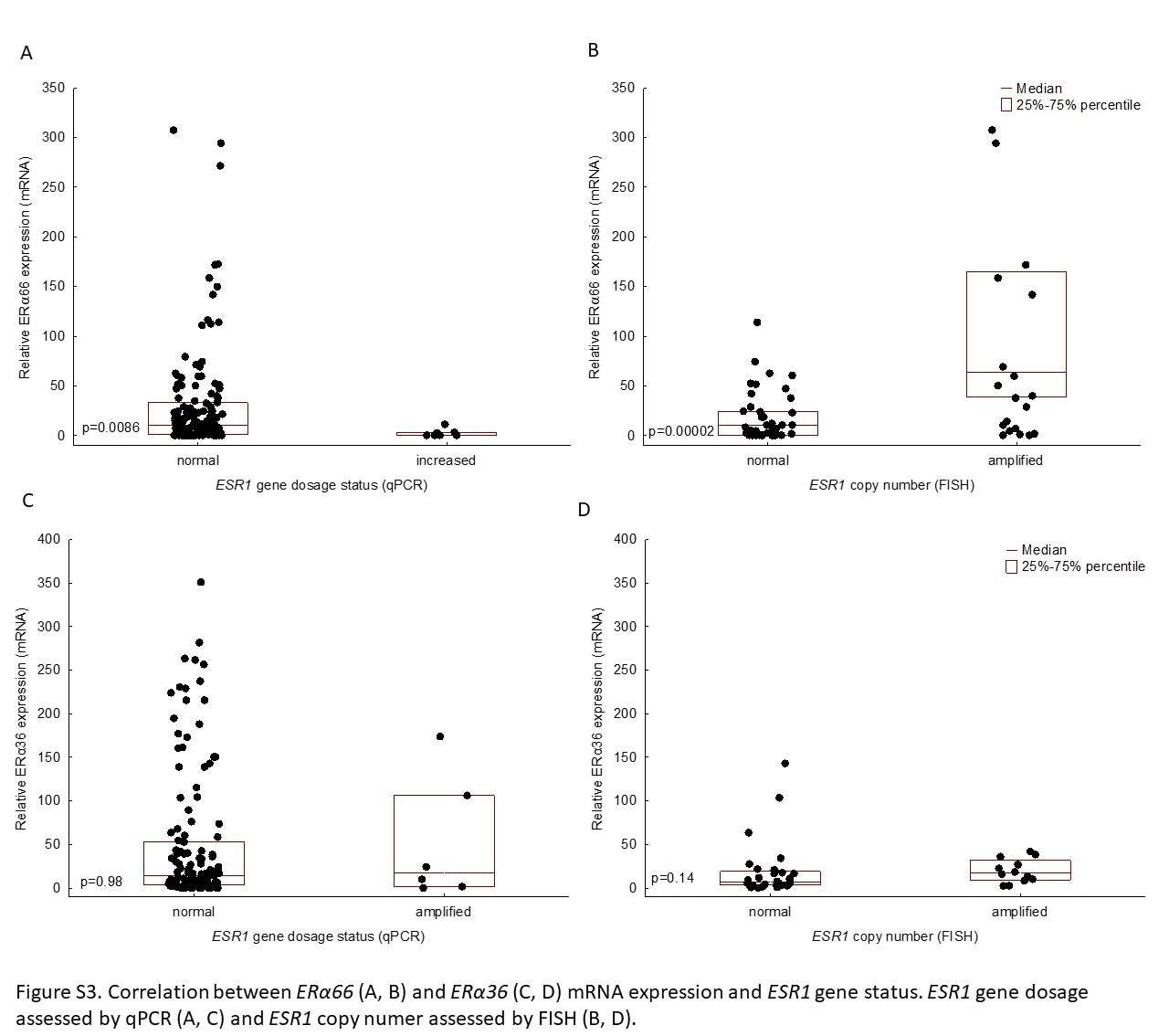

Supplement: Supplementary file 1 [file ijms-20-01881-s001.zip › Supplemantary data/Figure S3.jpg]

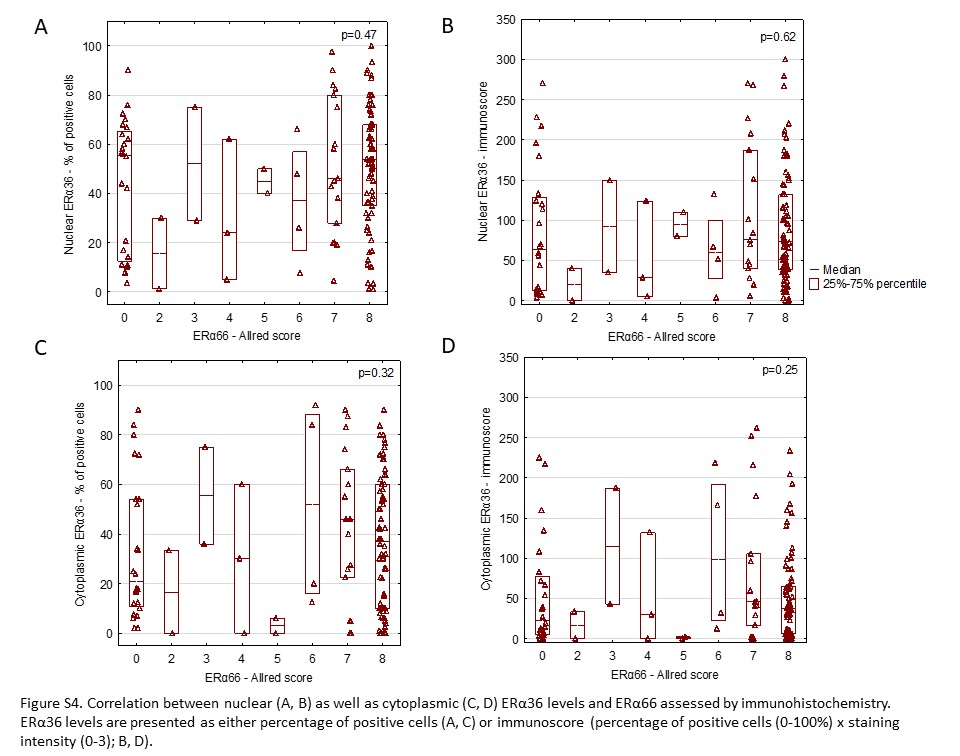

Supplement: Supplementary file 1 [file ijms-20-01881-s001.zip › Supplemantary data/Figure S4.jpg]

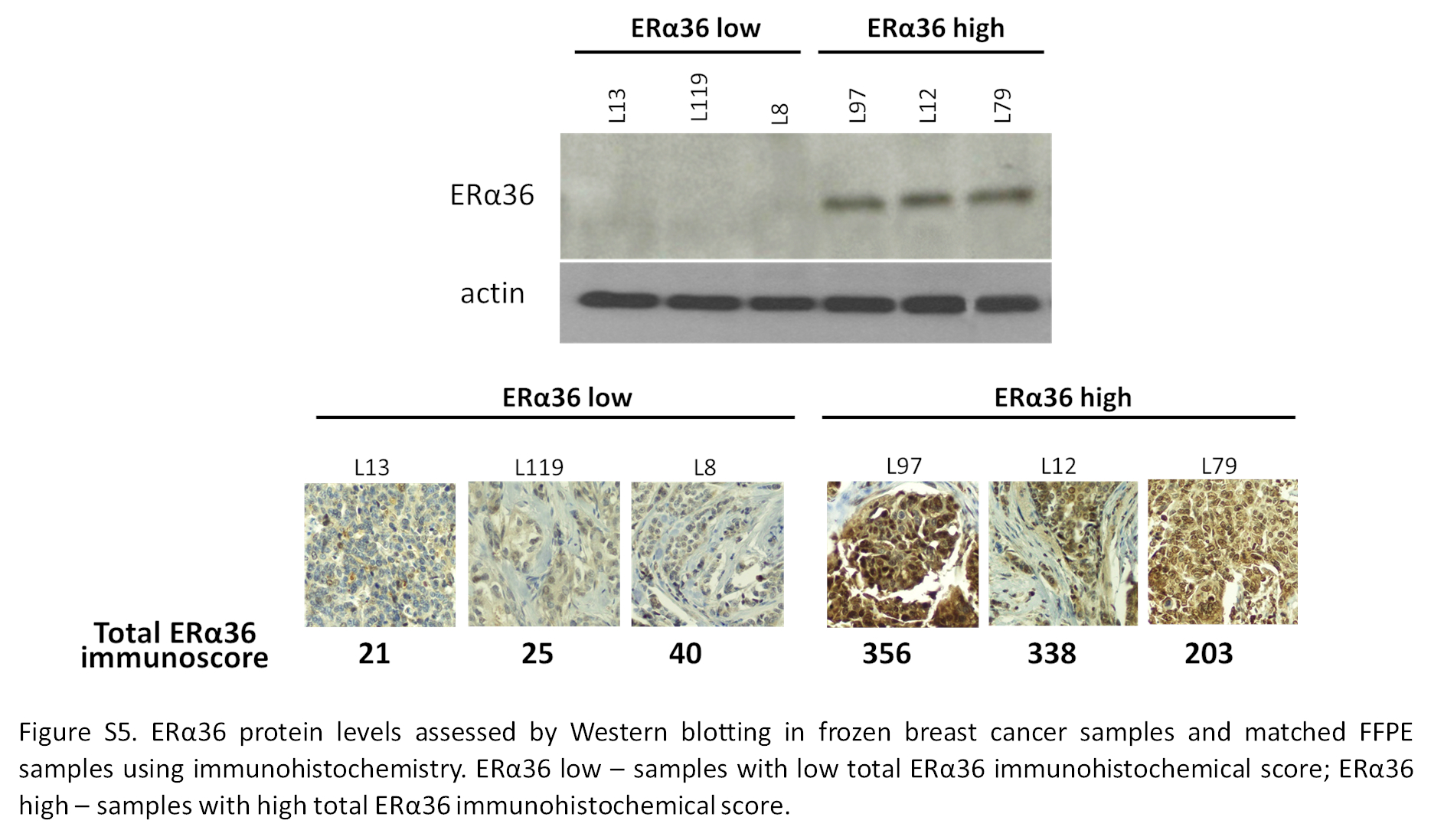

Supplement: Supplementary file 1 [file ijms-20-01881-s001.zip › Supplemantary data/Figure S5.jpg]
